# Analysing seasonal rainfall trends in the Cuvelai-Etosha Basin 1968–2018

**DOI:** 10.4102/jamba.v17i1.1654

**Published:** 2025-03-10

**Authors:** Buhlebenkosi F. Mpofu, Nnenesi Kgabi, Stuart Piketh

**Affiliations:** 1 Unit of Environmental Sciences and Management, Faculty of Natural and Agricultural Science, North West University, Potchefstroom, South Africa

**Keywords:** climate change, rainfall, trends, resilience, seasons, floods, water

## Abstract

This research used descriptive statistics to analyse rainfall trends in the Cuvelai- Etosha Basin (Namibia) over a 50-year historical period (1968 to 2018). The results revealed that rainfall fell over a period of 6 months between the months of November and April. Rainfall amounts were also observed to be higher in the first 3 months of each year, and annual levels ranged between 200 mm and 700 mm. The trend revealed that rainfall levels between 1977 and 1992 were consistently below the calculated average of 410 mm, and the rainfall amounts, and rain season were observed to have significantly shortened between the years 2009 and 2018. The rainfall trend observed over the 50-year period did not provide a definitive indication of whether the pattern followed a specific trajectory. The trend line’s position was below the average line for many seasons, and it indicated that many of the seasons experienced rainfall levels below the annual average; however, an increase was observed from the years 2008 -2012 and the year 2018 wherein the rainfall received was above average and fell intensely over a brief period and these are the years where flooding was reported.

**Contribution:**

An epileptic pattern was observed that could not be used to definitively define a trend but was useful to highlight that there was an occurrence of episodes of heavy rainfall being experienced in the months of January through March and any resilience efforts need to be prioritised during this time.

## Introduction

Rainfall is an important element of the earth’s climate system and it influences water resources, ecosystems, agriculture and human livelihoods depend on it. Rainfall trends vary around the world, with some regions experiencing increased rainfall intensity and frequency while others face prolonged drought leading to a wide range of consequences such as flooding and water scarcity (Tamm et al. 2023). The study of atmospheric processes is important in understanding rainfall distribution, variability, and its implications on patterns on regional and global scales (Trenberth 2011). Advancements in meteorological studies are useful in explaining how and why rainfall occurs, through study of air masses, condensation and water vapour and development of clouds (Monir et al. 2023). Furthermore, meteorological studies have enabled the detection of rainfall changes and also to assess the drivers and model potential future scenarios (Sokol et al. 2021).

Rainfall data is used for different purposes in planning, research and practice. Real time daily measurements can be used for decision making in town planning, agriculture productivity, climate change analysis, water resource management and may be used to estimate flooding in some geographical areas (Harsha 2017). Real time rainfall data refers to rainfall data that are available from database daily and sent through electronic means for utilisation (Morbidelli et al. 2021). It can be analysed historically or in real time to check for rainfall amounts, frequency and intensity. Historical records are useful in analysing trends and pattern and can be used to anticipate drought and flood conditions. According to Rogers (2020), a combination of techniques are used to measure rainfall and each has its own advantages and disadvantages. Traditionally, rainfall is measured at a weather station using rain gauges such as tipping buckets that measure rainfall using a tipping mechanism, where each tip is recorded by a data logger. Current new technologies are now complementing the traditional rain gauge with radar technology, satellite imagery and interpolation techniques to estimate rainfall over a spatial surface (Auguste 2018; Carvalho 2014). Rainfall measurement in Africa is important in the support of sectors of the economy but more so, in agriculture as it forms the backbone of the most economies. The primary source of water in agricultural production for most of Africa is rainfall and if there are any fluctuations in rainfall availability, it may impact agricultural productivity and revenue, leaving communities vulnerable and likely food insecure (Alahacoon et al. 2022; Olanrewaju 2019).

Research on rainfall seems to depict more uncertainties in the future for the world at large. The predictions are that there will be tangible changes in rainfall patterns, but what is uncertain is the nature of these changes and whether there will be decreases or increases in rainfall and at what intensity (Huddleston 2016). Understanding rainfall uncertainties is important because rainfall is a key driver of floods, droughts and plays an important role in the global water cycle (Kundzewicz 2016; Vicente-Serrano& Li 2021). Rainfall, extremes can either be intense (leading to flooding) or lack (leading to droughts) and they are defined as unexpected, unusual, severe and unseasonal phenomenon that cause damage, destruction and sometimes even loss of life (Huber & Gulledge 2011). Africa, for instance, has been prone to prolonged droughts and spatio-temporal variations in rainfall regimes (Franchi et al. 2024). Recent rainfall data sets depict negative trends especially in sub-Saharan Africa (Harrison, Funk & Peterson 2019), which unfortunately will have a negative bearing on the livelihoods of communities that rely on agriculture for subsistence. According to Kotir (2011), sub-Saharan Africa has been highlighted as a vulnerable region because of its dependence on rainfed agriculture, erratic rainfall, and its low capacity to adapt. At a global level, scientists predict that extreme weather events, will increase in frequency and the environment will become more sensitive to extreme weather because of population growth in areas more vulnerable to weather and climate extremes (Mika 2011).

The Cuvelai-Etosha Basin is a natural transboundary wetland that is shared by Angola and Namibia and is homeland to many communities (Mendelsohn & Beat 2011). It is characterised by the presence of shallow groundwater and relatively fertile soils. Kolberg and Simmons (1996) state that the Cuvelai drainage is an important system as it is a naturally occurring wetland that supports local water resources as well as community livelihoods in the area. The Cuvelai Basin has experienced and continues to experience extensive flooding, with negative repercussions including the loss of human lives and property (Hango 2017). In 2009, for example, the floods displaced approximately 13 000 people from different communities and left 92 people dead (Kotir 2011). In other years, the floods have destroyed crops and interfered with livelihoods, thus putting the region at risk of food shortages amongst other consequences (Allen 2009).

Climate projections of the area predict extremes of temperature, evaporation and variable rainfall, and these are likely to worsen the challenges that Namibia is already facing as an arid country (Wilhelm 2012). To be able to ensure adaption and resilience to flooding, it is necessary to have timeous information as this will strengthen the adaptive capacity and in turn, scale up adoption of coping mechanisms and measures, thus ensuring that communities become resilient.

The main purpose of this study was to analyse precipitation trends in the Cuvelai-Etosha Basin in Namibia over a 50-year period from 1967 to 2018. The primary objective was to identify rainfall trends and determine whether any observable patterns exist, while also pinpointing seasons of significant rainfall. The research question associated with this objective was: ‘What precipitation patterns have been experienced over the last 50 years?’ The corresponding hypothesis stated: ‘There are distinct precipitation patterns and trends in the Cuvelai-Etosha Basin over the last 50 years, showing significant variability in seasonal rainfall’. This led to the formulation of the following hypotheses:

- *Null Hypothesis (H_0_):* ‘There is no significant variability or change in precipitation patterns in the Cuvelai-Etosha Basin over the last 50 years’.
- *Alternative Hypothesis (H_1_):* ‘There is significant variability or change in precipitation patterns in the Cuvelai-Etosha Basin over the last 50 years’.

## Research methods and design

For the analysis of rainfall data, Statistical Package for Social Sciences (SPSS) was used for data cleaning, normality and hypothesis testing. The choice to use SPSS was motivated by the fact that the software has the ability to handle and process large data sets without significant computational delays. Furthermore, SPSS has a built-in time series analysis function that allows researchers to model and evaluate trends over time (Masuadi et al. 2021).

### Description of the study area

Oshakati Town is one community affected by floods. It is located at latitude 17°46′59.99′S and longitude 15°40′59′.9′E in northern Namibia and was founded in July 1966 (Van Zyl et al. 2012). The flood history of this town specifically highlights 2008, 2009, 2010, 2011 and 2018 being the years that brought floods into the area.

Monthly and daily rainfall data for this period were obtained from the archives of the Namibian Meteorological Service (NMS) and the Ministry of Agriculture, specifically from the Oshakati and Ondangwa weather stations. Because the rainfall data were over a 50-year period, it was anticipated that data quality issues might arise and, therefore, the research incorporated data-wrangling steps to address the issues. The wrangling steps involved treating missing data, conducting reliability testing, and shaping the data to ensure that it was suitable for its intended use as described next.

### Treatment of missing data

The data were analysed for continuity to check for any missing data, and it was noticed that there was missing rainfall data for a 10-year period between 1993 and 2003. The data obtained from both weather stations were consistent, and there were no other records in the archives specifically for the study area. Consequently, the researcher decided to divide the data into two subsets: subset 1 covering the years 1968 to 1993 and subset 2 covering the years 2004 to 2018. Thus, a total of 40 years were investigated. Furthermore, a missing value analysis on the daily recordings indicated that 0.6% of the data were missing, and these missing values were coded as either -99999 or ***** on the raw datasheet. For data completeness, this research performed interpolation of Oshakati data with the Ondangwa data, reducing the proportion of missing values to 0.41%.

### Reliability of data

The analysis involved various statistical measures, including the mean, median, kurtosis and skewness. The mean and median values were found to be in proximity, with values of 39.37 and 39 days, respectively. The close alignment between the mean and median indicated that the data followed a symmetric distribution, typical of a normal distribution. The similarity between these central tendency measures further suggested that the data points are evenly distributed around the central value. In addition, the kurtosis and skewness values are computed to be −0.0872 and −0.0019, respectively. Both values are very close to zero, which is another indication that the data are normally distributed. A kurtosis value close to zero implies that the distribution has a similar shape to the standard normal distribution (mesokurtic) and a skewness value near zero signifies that the data are nearly symmetric around the mean.

While the normal test on the number of precipitation days yielded positive results, it was observed from the findings that there are months with an insignificant contribution to the phenomenon being investigated and some years even had missing monthly data, amounting to approximately 11.6% of the total data. As this figure is slightly double the acceptable threshold of 5%, the research made two decisions to address this issue.

#### Pruning months with less than 5% contribution

The research decided to remove the months that have a contribution of less than 5%. The rationale behind this decision follows the assumption made by Little and Rubin (2019) that data representing less than 5% have an insignificant impact on the results of the investigation. Therefore, it is reasonable to discard data from such months.

#### Adherence to official rainy season

The research also decided to adhere to the official definition of the rainy season for the area of study. This means that only data from the months considered part of the official rainy season would be retained for further analysis. Therefore, the months of May through October were not considered part of the official rainy season.

After implementing the decisions to prune the months with less than 5% contribution and sticking to the official rainy season, the proportion of missing data significantly reduced from 11.6% to 4.1%. This reduction in missing data improved the overall quality and reliability of the analysis. In addition, to organise the data and facilitate the analysis, the research grouped the data into seasons. For instance, a season like ‘1968’ includes data from November and December of the preceding year (e.g. 1967) and data from January to April of 1968. This season grouping method helped in considering a broader timeframe and captured relevant patterns across multiple months. As a result of this data organisation, the total number of seasons under investigation was 40.

### Ethical considerations

Ethical clearance to conduct this study was obtained from the North-West University Faculty of Natural and Agricultural Sciences Ethics Committee (No. NWU-00471-21-A9) and Namibia Council of Research, Science and Technology (No. RPIV01042031). Research was conducted in compliance with relevant laws, regulations and ethical guidelines. There were no human participants in this study. The study is also part of the graduate studies programme.

## Results and discussion

### Analysis of precipitation data

**FIGURE 1:**
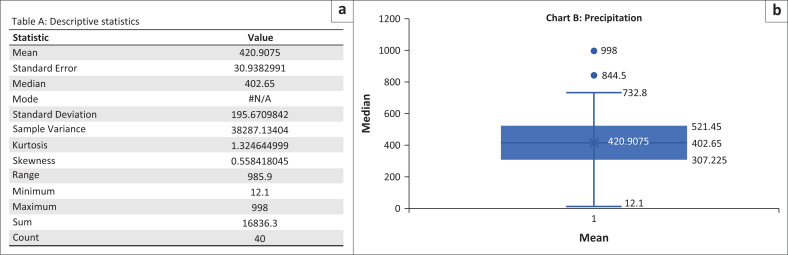
Data normality test results for precipitation data. a) Descriptive statistics; b) Box and whisker plot.

Descriptive statistics for the precipitation data over the period under review indicated that the data closely followed a normal distribution. The mean and median values were not significantly different, suggesting a symmetrical distribution (Figure 1) around the central tendency.

Figure 1 presents the descriptive statistics and a Box and Whisker chart for the precipitation data in Table 1 and Chart B, respectively. The mean and median values are 420.9 mm and 402.65 mm per season, respectively, and they are not significantly different. The data show a slight positive skewness of approximately 0.558, which is within the acceptable normal standard range of ±1.5. The kurtosis is also within the acceptable range at approximately 1.324, as highlighted in Table 1.

**TABLE 1:** Summary of precipitation data organised in seasons.

Season	Month (mm)						Season total (mm)	Season contribution	stdev	Mean
	Nov	Dec	Jan	Feb	Mar	Apr				
1968	258.8	153.8	69.6	37.8	195.6	17.2	**732.8**	4.4	95.88	122.13
1969	80.0	51.2	140.8	158.8	29.0	30.4	**490.2**	2.9	56.18	81.70
1970	60.0	17.5	89.4	69.1	42.0	5.0	**283.0**	1.7	31.98	47.17
1971	15.5	22.8	178.6	253.0	109.5	0.0	**579.4**	3.4	102.67	96.57
1972	1.7	84.0	150.5	15.5	168.1	0.0	**419.8**	2.5	75.94	69.97
1973	1.0	18.3	58.7	13.4	171.5	92.5	**355.4**	2.1	64.62	59.23
1974	0.0	0.0	219.2	202.3	187.0	38.5	**647.0**	3.8	105.51	107.83
1975	25.5	62.5	127.2	53.8	135.5	46.8	**451.3**	2.7	45.25	75.22
1976	38.8	5.5	177.9	246.5	186.0	21.0	**675.7**	4.0	102.85	112.62
1977	57.9	9.0	124.3	164.5	20.7	26.0	**402.4**	2.4	63.34	67.07
1978	8.5	0.0	0.0	0.0	0.0	3.6	**12.1**	0.1	3.49	2.02
1979	43.5	46.0	109.8	244.7	29.0	0.0	**473.0**	2.8	88.88	78.83
1980	55.3	43.7	49.9	131.9	113.0	0.0	**393.8**	2.3	48.53	65.63
1981	41.4	96.8	104.2	65.9	18.5	22.7	**349.5**	2.1	36.83	58.25
1982	6.6	9.9	91.1	98.4	134.3	27.2	**367.5**	2.2	53.65	61.25
1983	32.5	12.0	81.2	43.3	34.2	30.8	**234.0**	1.4	23.07	39.00
1984	0.0	54.5	66.0	121.4	136.0	22.9	**400.8**	2.4	53.50	66.80
1985	80.4	16.5	76.2	109.0	122.0	0.0	**404.1**	2.4	49.18	67.35
1986	28.5	59.5	171.7	70.7	157.2	28.0	**515.6**	3.1	63.28	85.93
1987	47.5	39.8	18.9	141.4	40.5	0.0	**288.1**	1.7	48.99	48.02
1988	28.2	7.5	29.5	6.5	12.5	0.0	**84.2**	0.5	12.15	14.03
1989	63.3	174.3	65.0	45.5	5.0	42.0	**395.1**	2.3	57.37	65.85
1990	0.0	0.0	156.0	109.0	73.5	0.0	**338.5**	2.0	67.12	56.42
1991	0.0	0.0	9.2	100.5	21.8	0.0	**131.5**	0.8	39.44	21.92
1992	0.0	16.4	77.2	11.7	0.0	0.0	**105.3**	0.6	30.06	17.55
2004	16.9	103.9	153.5	103.3	179.0	33.0	**589.6**	3.5	64.04	98.27
2005	136.6	1.7	154.7	93.0	0.0	0.0	**386.0**	2.3	72.68	64.33
2006	1.0	39.2	240.0	132.0	0.0	53.9	**466.1**	2.8	93.01	77.68
2007	23.3	10.9	100.2	48.7	78.6	24.3	**286.0**	1.7	35.24	47.67
2008	24.7	5.0	195.5	177.5	164.2	2.6	**569.5**	3.4	93.03	94.92
2009	73.4	77.5	213.2	378.4	94.4	7.6	**844.5**	5.0	134.27	140.75
2010	58.0	39.0	165.4	97.5	73.7	0.4	**434.0**	2.6	56.19	72.33
2011	119.7	85.4	221.1	243.6	246.4	81.8	**998.0**	5.9	79.06	166.33
2012	18.3	101.8	170.8	135.0	96.5	1.0	**523.4**	3.1	65.96	87.23
2013	55.6	100.6	36.6	18.2	92.8	0.0	**303.8**	1.8	40.28	50.63
2014	53.6	67.3	57.8	58.7	130.1	35.4	**402.9**	2.4	32.60	67.15
2015	30.4	63.3	95.0	40.8	64.4	23.6	**317.5**	1.9	26.55	52.92
2016	7.2	70.2	29.4	13.3	96.7	0.0	**216.8**	1.3	38.83	36.13
2017	142.6	115.2	41.1	190.3	40.2	28.6	**558.0**	3.3	66.41	93.00
2018	9.5	43.5	56.5	32.6	156.5	111.5	**410.1**	2.4	55.01	68.35
**Total**	**1745.7**	**1926.0**	**4372.9**	**4277.5**	**3655.9**	**858.3**	**16836.3**	**100.0**	**-**	**-**
**Month’s contribution (%)**	**10.37**	**11.44**	**25.97**	**25.41**	**21.71**	**5.10**	**100.00**	**-**	**-**	**-**
**average precipitation per month (mm)**	**49.88**	**53.50**	**112.13**	**109.68**	**101.55**	**31.79**	**420.91**	**2.50**	**59.32**	**67.35**

stdev, standard deviation.

The Box and Whisker chart illustrates that the average precipitation across the years appears relatively even, with little variation represented by the thinness of the box. The differences between the median and the upper and lower quartiles are approximately 118 mm (521.45 mm–402.65 mm) and 95.43 mm (402.65 mm–307.225 mm), respectively, indicating a normal distribution of the data.

**FIGURE 2:**
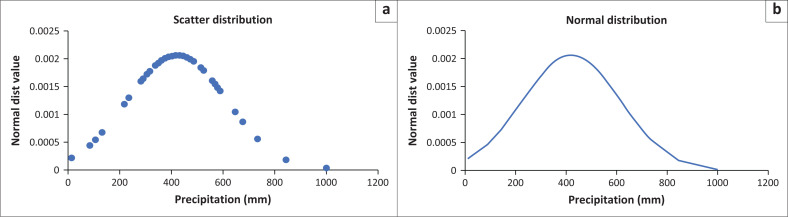
Normal probability plot and curve for precipitation data. a) Scatter distribution; b) Normal distribution.

Two outliers are observed beyond the whiskers on the Box and Whisker chart, with values of 998 mm and 844.4 mm, representing above-normal rainfall seasons for the years 2011 and 2009, respectively. A closer inspection confirms the accuracy of these values, requiring no further action. Moreover, when the precipitation data were transformed into normal distribution probability values and graphed, the resulting curve exhibits the characteristics of a normally distributed dataset, as shown in Figure 2.

Figure 2 presents two representations of the precipitation data, Chart A in scatter form and Chart B in curve form, both displaying the normal distribution plots. The horizontal axis represents precipitation measured in mm, while the vertical axis represents the associated normal probability values. In Chart A, the density of the plot points is highly concentrated around the peak of the curve, confirming that most of the years have their total precipitation centred around the mean value of approximately 400 mm. Connecting these plot points results in a somewhat smooth curve, as seen in Chart B. However, a slight tail is visible on the curve because of the presence of two outliers of 998 mm and 844.5 mm of precipitation.

The overall shape of the curve in Chart B indicates that the data closely follow a normal distribution pattern, with most of the data points clustered around the mean, representing the typical precipitation levels experienced across the years. The descriptive statistics, Box and Whisker chart, and the normal distribution probability graph collectively provide strong evidence supporting the conclusion that the precipitation data adhere to a normal distribution pattern. This confirmation of normality is essential for ensuring the validity of statistical analyses, modelling, and making accurate predictions based on the data.

The analysis of precipitation data spanning 40 years reveals significant variations in the amount of precipitation received across different months. January, February and March stand out as the months receiving the highest amounts of precipitation, contributing 25.97%, 25.42% and 21.71%, respectively, to the total precipitation over the entire period. In contrast, April’s contribution is notably lower, representing only 5.1% of the total precipitation. This is summarised in Table 1.

Table 1 provides a comprehensive summary of the total precipitation received for each month during the 40-year period, organised into seasons. The ‘Season Total’ column presents the cumulative amount of precipitation, representing the sum of precipitation for the months in columns 2 through 7. The ‘Season’s Contribution’ column expresses this value as a percentage, indicating the proportion contributed by each season to the total precipitation over the 40-year period. The last two columns display the standard deviation and mean precipitation values on a seasonal basis. The standard deviation serves as an indicator of how the data are clustered around the mean. Seasons with higher standard deviations, such as 1971, 1974, 1976 and 2009, exhibit greater data spread, which explains the presence of some outliers in the dataset. As a result, this research assumed that the probability of floods is higher during these seasons because of the likelihood of extremely elevated precipitation levels.

Further scrutiny of the data reveals that the seasons of 2011 and 2009 received the highest precipitation recording 998 mm and 844.5 mm, respectively. Conversely, the season of 1998 experienced the least amount of precipitation, totalling only 84 mm. Interestingly, this amount is even lower than the mean monthly precipitation of other seasons such as 2009, 2011 and 1976, among others. For the season of 1978, a closer examination revealed that records were unavailable for 4 out of 6 months, possibly impacting the representation of data for that season.

Among the months within the rainfall season, January, February and March stand out as the most wet, with average monthly precipitation of approximately 112.13 mm, 109.68 mm and 101.55 mm, respectively. In addition, the seasons from 2009 to 2018 are recorded to have experienced floods in the community of Oshakati, Ehenye community (Hango 2017). However, this does not infer that other seasons are flood-free. The main reason why there are no flood records for other seasons is because the study area was un-inhabited pre-2007; as such, none was affected by the floods.

**FIGURE 3:**
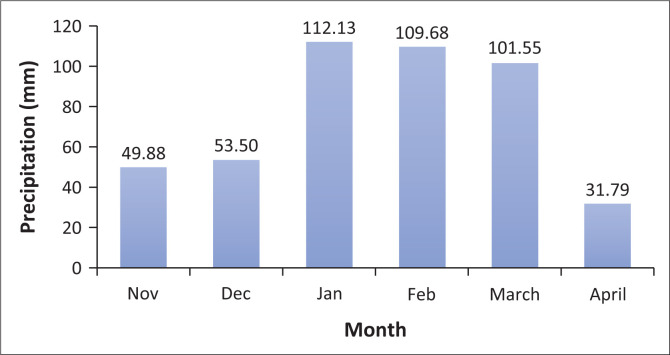
Monthly average precipitation.

For further visualisation, refer to Figure 3, where the graphical representation of these monthly averages is presented.

Figure 3 depicts the monthly average precipitation over the period, with the horizontal axis representing the months and the vertical axis indicating precipitation in mm. A notable observation from the graph is the intensified precipitation experienced during a 3-month period from January through March. Based on this finding, the research suggests that these 3 months should be the primary focus for any resilience efforts, considering their higher precipitation levels.

By utilising the monthly average precipitation data from Figure 3 and combining it with the average number of precipitation days per month, this research successfully computed the approximate level of precipitation per day. The results of this computation are summarised in Table 2. This information allows for a more granular understanding of the daily precipitation patterns, which is valuable for various applications, such as planning and preparedness for weather-related events.

**TABLE 2:** Average precipitation per day of the month.

Precipitation metric	Nov	Dec	Jan	Feb	Mar	Apr	Seasons average
Avg precipitation per month (mm)	49.88	53.50	112.13	109.68	101.55	31.79	420.91
Aavg no of precipitation days	5.00	5.40	9.10	8.80	7.60	2.10	38.00
Avg precipitation per day (mm)	9.98	9.91	12.32	12.46	13.36	15.14	11.08

Table 2 provides a summary of the approximate level of precipitation for each precipitation day across different months. Interestingly, while January and February have the highest average precipitation values, it is the month of April, followed by March, that experiences higher daily precipitation levels, averaging approximately 15.14 mm and 13.36 mm, respectively. This observation is significant because it implies that even though April has fewer precipitation days (only 2.1 days on average), the rainfall levels on those days are relatively higher. Consequently, this could lead to a higher risk of flash floods during those short rainy periods. According to Fowler, Wasko and Prein (2021), rainfall intensity occurrence plays a role in inducing flooding especially in areas that are ill equipped to manage rapid water accumulation.

Moreover, the fact that April experiences fewer rainy days may also lead to the likelihood of a prolonged dry spell, which can have implications for farmers and agriculture in the region. The onset of a rainy season in a semi-arid climate as defined by Seregina et al. (2019), is rainfall that exceeds 5 mm for 3 consecutive days or more. The rainy season in Namibia is clear and easy to identify as seasons are clearly defined and according to the rainfall data, the rainy season is from November to April although there are a few outliers in other months such as October and May. Average annual rainfall can range from 200 mL to 700 mL. Heavy rainfall is defined as 100 mm of rainfall in 24 h or less for 3 days or more (Zambuko 2011). In the 40 years of analysed rainfall data, the month of January had the highest rainfall record and was followed by the months of February and March, respectively. Less rain was recorded in the months of November and April.

### Precipitation trend analysis

**FIGURE 4:**
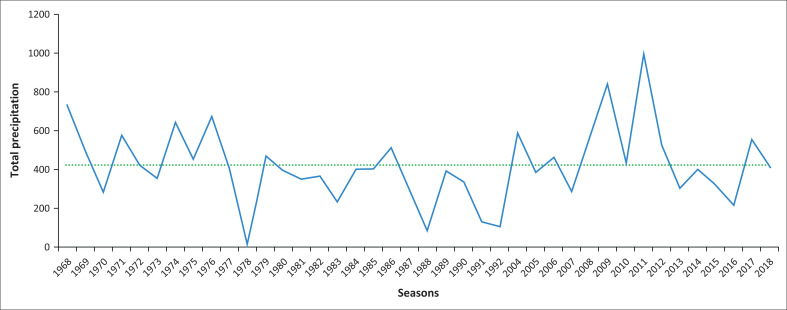
Precipitation trend line.

The precipitation trend observed over the 40-year period does not provide a definitive indication of whether the rainfall pattern is following a specific trajectory. However, the trend graph does reveal that the precipitation levels from 1977 to 1992 were consistently below the average of 410 mm, save for seasons 1979 and 1986 as depicted in Figure 4.

The depiction of the precipitation trend in Figure 4 shows the variations in total precipitation across seasons over the observed period. The horizontal axis represents the seasons and the vertical axis represents the total precipitation per season. The green dotted line represents the average precipitation, while the blue line represents the overall trend.

The trend line’s position below the average line for many seasons indicates that many of the seasons experienced precipitation levels below the average annual rainfall. This suggests a pattern of lower-than-average rainfall over the observed period. However, it is important to notice that while the trend line provides valuable historical information about the seasonal precipitation patterns, it might not be sufficient to predict future seasonal precipitation accurately. Predicting future weather patterns and rainfall with certainty is challenging because of the complexity and variability of climate systems. To make more accurate predictions about future seasonal precipitation, additional factors and sophisticated models, such as climate models, should be considered. These models consider various climatic influences, atmospheric conditions and other variables to provide more reliable forecasts. Nonetheless, the trend line in Figure 4 serves as a valuable representation of the observed historical patterns of seasonal precipitation without the fibre temporal resolution just to indicate variations in annual rainfall patterns. According to Adler et al. (2017), knowing the rainfall trends globally and how they vary is important for understanding water-related resources that are used in the sustenance of human lives, as well as various ecosystems and other critical parameters. The amount of rainfall at a given time can have wide ranging effects on the environment in general as well as on human lives and how they function (Trenberth 2011). Any small changes in rainfall can cause disruption to a wide range of natural processes especially when changes occur quickly; therefore, it is important to pay attention to rainfall trends so that any anomalies and/or differences beyond the known average can be closely monitored as they may have impact on living things (Arnbjerg-Nielsen et al. 2013). Investigations into the changing trends of temperature and precipitation are important as they underpin adaptive strategies of human ecosystems and water resource planners (El-ashry & Zeidler 2009; Niang et al. 2015).

### Hypothesis test results

A linear regression analysis was conducted to examine the trend in precipitation data from 1968 to 2018 in the Cuvelai-Etosha Basin, Namibia. The regression model assessed the relationship between year (independent variable) and annual precipitation (dependent variable), aiming to determine if there was a significant trend over the period. The results of the regression analysis (Table 3) show that the relationship between year and precipitation is not statistically significant, with a *p*-value greater than 0.05. The slope coefficient for year was 1.506, suggesting a slight increase in precipitation over time, but this increase is not meaningful given the high *p*-value. The R-squared value was very low, indicating that the model explains little of the variance in the precipitation data. The variability in precipitation over the years was also assessed. The mean annual precipitation over the period was 420.91 mm, with a standard deviation of 195.67 mm, indicating considerable year-to-year variation. The precipitation ranged from 12.1 mm to 998.0 mm, with 50% of the precipitation values falling between 314.08 mm and 517.55 mm.

**TABLE 3:** Regression analysis summary for precipitation vs. year.

Coefficients	B	SE	*t*	*p*	95% Confidence interval
Constant	−2597.65	1870.59	−1.39	0.17	[-6413.94, 781.353]
Year	1.51	0.94	1.61	0.12	[-0.40, 3.41]

B, beta coefficient; SE, standard error; *t, t*-statistic; *p, p*-value.

The analysis in Table 1 shows that there is no significant trend in precipitation patterns over the period in the Cuvelai-Etosha Basin. The *p*-value of 0.117 indicates that we fail to reject the null hypothesis, meaning that any observed trend in the data is likely because of random variation rather than a significant long-term change. In addition to the trend analysis, the descriptive statistics show considerable variability in precipitation from year to year, with a standard deviation of 195.67 mm and values ranging from 12.1 mm to 998.0 mm. These results suggest that while there is variability, there is no consistent pattern of increase or decrease over time.

## Conclusion

Based on the statistical tests conducted, it was observed that the rainfall amounts received by Oshakati were generally low, although there were a few years with intense rain over short periods. According to the analysis, the amount of rainfall received was not substantial enough to cause significant alarm. Despite this, the reason behind the recurrent flooding in the Oshakati community remained unresolved. In summary, the results indicated no significant variability in rainfall, except for the year 2011 and this was not significant enough to explain the frequent flooding in the area. The study was handicapped by lack of data for 10 years between 1993 and 2003 and thus affected the generation of the complete 50-year trend. Despite this, it was clear that the years 2009 and 2011 were the ones that received above-average rainfall leaving the question of the cause of recurrent flooding unanswered.

### Recommendations

Further investigations need to be conducted to establish why there is recurrent flooding in Oshakati. The investigations could consider a physical and infrastructural analysis to establish if there are any predisposing factors that could contribute to the flooding in the area.
